# A rule-based electronic phenotyping algorithm for detecting clinically relevant cardiovascular disease cases

**DOI:** 10.1186/s13104-017-2600-2

**Published:** 2017-07-14

**Authors:** Santiago Esteban, Manuel Rodríguez Tablado, Ricardo Ignacio Ricci, Sergio Terrasa, Karin Kopitowski

**Affiliations:** 1 Family and Community Medicine Division, Hospital Italiano de Buenos Aires, Tte. J. D. Peron, 4272 Buenos Aires, Argentina; 2 Research Department, Instituto Universitario del Hospital Italiano de Buenos Aires, Buenos Aires, Argentina

**Keywords:** Cardiovascular disease, Cerebrovascular disease, Electronic phenotyping algorithms, Electronic medical records, Rule-based algorithm

## Abstract

**Background:**

The implementation of electronic medical records (EMR) is becoming increasingly common. Error and data loss reduction, patient-care efficiency increase, decision-making assistance and facilitation of event surveillance, are some of the many processes that EMRs help improve. In addition, they show a lot of promise in terms of data collection to facilitate observational epidemiological studies and their use for this purpose has increased significantly over the recent years. Even though the quantity and availability of the data are clearly improved thanks to EMRs, still, the problem of the quality of the data remains. This is especially important when attempting to determine if an event has actually occurred or not. We sought to assess the sensitivity, specificity, and agreement level of a codes-based algorithm for the detection of clinically relevant cardiovascular (CaVD) and cerebrovascular (CeVD) disease cases, using data from EMRs.

**Methods:**

Three family physicians from the research group selected clinically relevant CaVD and CeVD terms from the international classification of primary care, Second Edition (ICPC-2), the ICD 10 version 2015 and SNOMED-CT 2015 Edition. These terms included both signs, symptoms, diagnoses and procedures associated with CaVD and CeVD. Terms not related to symptoms, signs, diagnoses or procedures of CaVD or CeVD and also those describing incidental findings without clinical relevance were excluded. The algorithm yielded a positive result if the patient had at least one of the selected terms in their medical records, as long as it was not recorded as an error. Else, if no terms were found, the patient was classified as negative. This algorithm was applied to a randomly selected sample of the active patients within the hospital’s HMO by 1/1/2005 that were 40–79 years old, had at least one year of seniority in the HMO and at least one clinical encounter. Thus, patients were classified into four groups: (1) Negative patients (2) Patients with CaVD but without CeVD; (3) Patients with CeVD but without disease CaVD; (4) Patients with both diseases. To facilitate the validation process, a stratified sample was taken so that each of the groups represented approximately 25% of the sample. Manual chart review was used as the gold standard for assessing the algorithm’s performance. One-third of the patients were assigned randomly to each reviewer (Cohen’s kappa 0.91). Both coded and un-coded (free text) sections of the EMR were reviewed. This was done from the first present clinical note in the patients chart to the last one registered prior to 1/1/2005.

**Results:**

The performance of the algorithm was compared against manual chart review. It yielded high sensitivity (0.99, 95% CI 0.938–0.9971) and acceptable specificity (0.86, 95% CI 0.818–0.895) for detecting cases of CaVD and CeVD combined. A qualitative analysis of the false positives and false negatives was performed.

**Conclusions:**

We developed a simple algorithm, using only standardized and non-standardized coded terms within an EMR that can properly detect clinically relevant events and symptoms of CaVD and CeVD. We believe that combining it with an analysis of the free text using an NLP approach would yield even better results.

**Electronic supplementary material:**

The online version of this article (doi:10.1186/s13104-017-2600-2) contains supplementary material, which is available to authorized users.

## Background

Over the last 30 years, electronic medical records (EMR) are becoming a standard tool of care in both in ambulatory and inpatient settings. Error and data loss reduction, patient-care efficiency increase, decision-making assistance and facilitation of event surveillance are some of the many processes that EMRs help improve [1, 2]. In addition, they show a lot of promise in terms of data collection to facilitate observational epidemiological studies and their use for this purpose has increased significantly over the recent years [3–5].

Even though the quantity and availability of the data for research are clearly improved thanks to EMRs, still, the problem of data-quality remains. This is especially important when attempting to determine if an event has actually occurred or not. In case of having a problem-based EMR, the determination of the events can be performed through the selection of combinations of coded terms (diagnoses, procedures, etc.), and also through the development of algorithms which use the data contained in free-text clinical notes [3, 6]. This can be implemented towards determining the occurrence of events [7–22], but also in order to define exposure to risk factors [23–31] (i.e., diabetes, smoking status, etc.), medication use [32–37] and monitoring adverse events [38–40]. This process is usually referred to as electronic phenotyping.

The Hospital Italiano de Buenos Aires (HIBA, Italian Hospital of Buenos Aires) has developed and implemented an in-house EMR since the year 2000, and currently allows registration of outpatient, home-care, inpatient and emergency-room episodes. The EMR’s terminology system is based on the systematized nomenclature in medicine (SNOMED-CT). It is a flexible system that allows for standardized and also customized terms to be added. These terms are then linked to the clinical notes that are stored in free text format.

Several studies have described algorithms to detect events such as acute myocardial infarction (MI) [13, 41–44], stroke [17, 45], or chronic problems, such as congestive heart failure (CHF) [13, 22, 43, 46–50] or peripheral vascular disease (PVD) [11]. Nevertheless, because of both heterogeneity in medical practice and language barriers, these algorithms are dependent on the context they were developed on and usually need local adaptations in order to achieve high classification accuracy. Thus, we decided to locally develop and validate an algorithm for the detection of clinically relevant CVD, based on diagnostic codes from the EMR at HIBA in any clinical setting (inpatient, outpatient and emergency room visits).

## Objectives

To assess the sensitivity, specificity, and agreement level of a rule-based algorithm for the detection of cardiovascular and cerebrovascular disease, using structures data from electronic medical records.

## Methods

### Algorithm development

Three family physicians from the research group (SE, RIR, MRT) selected clinically relevant Cardiovascular disease (CaVD) and Cerebrovascular disease (CeVD) terms from the international classification of primary care, Second Edition (ICPC-2), the ICD 10 version 2015^[§]^ and SNOMED-CT 2015 Edition. These terms included both signs, symptoms, diagnoses and procedures associated with CaVD and CeVD. Using these terms as reference, we run a query in the HIBA’s EMR database^[††]^. This yielded a list of codes that was revised and agreed upon by the three reviewers. Terms not related to CaVD or CeVD symptoms, signs, diagnoses or procedures were excluded. Terms describing incidental findings without clinical relevance, were also excluded. Discrepancies were resolved in favor of maximizing the algorithm’s sensitivity. A list of the SNOMED-CT codes included in the final algorithm can be found in Additional file 1: Appendix. The algorithm yielded a positive result if the patient had at least one of the selected terms in their medical records, as long as it was not recorded as an error. Else, if no terms were found, the patient was classified as negative.

The algorithm was applied to a randomly selected sample of the active patients within the hospital’s HMO by 1/1/2005 that were 40–79 years old, had at least one year of seniority in the HMO and at least one clinical encounter. Thus, patients were classified into four groups: (1) Negative patients (2) Patients with CaVD but without CeVD; (3) Patients with CeVD but without disease CaVD; (4) Patients with both diseases. To facilitate the validation process, a stratified sample was taken so that each of the groups represented approximately 25% of the sample.

### Manual review of medical records

Three family physicians (SE, RIR and MRT) participated in this process. One-third of the patients were assigned randomly to each reviewer. Both coded and un-coded (free text) sections of the EMR were reviewed. This was done from the first clinical found note in the patients’ records to the last one registered prior to 1/1/2005.

### Manual classification criteria

We considered a case as positive, if positive mentions regarding CaVD or CeVD medical history, signs, symptoms, procedures or treatments were found in their records (inpatient, outpatient or emergency room clinical notes). Regarding results from diagnostic tests or procedures, in order to focus only on clinically relevant pathology, we only classified as positive those patients in whom positive test results fulfilled one of the following two conditions: (1) the result was preceded by some CaVD or CeVD compatible clinical context; (2) that, even though the result detected an incidental finding (i.e. electrocardiographic findings of an MI without symptoms), treatment was prescribed which would mean that the treating physician attributed some degree of clinical relevance to it. Also, we classified as positive, codes registered prior to the implementation of the EMR, assuming that it was any part of the patient’s past medical history.

On the other hand, we considered that the patient did not have CaVD or CeVD if: (1) There was no mention in the clinical notes of CaVD or CeVD; (2) A health problem related to CaVD or CeVD was mentioned but later on dismissed (even if this rejection was made after 1/01/2005); (3) A diagnostic code related to CaVD or CeVD was recorded, but there was no record of it in the free text of the clinical notes. An example of this are diagnostic codes that were recorded in the patient’s EMR, but did not represent a clinical entity but rather an incidental finding (i.e., vascular lesions detected in a brain CT scan that had not been ordered in the context of neurological symptoms). Also, we classified the finding as incidental if it did not trigger any preventive or therapeutic measures.

Unclear cases were subject to discussion between the three reviewers. The level of agreement between reviewers was estimated from a sample of 50 records using the Cohen’s κ statistic. The yielded value was 0.91^[‡‡]^.

### Quantitative analysis

The operational characteristics (sensitivity, specificity, agreement level with 95% confidence intervals) of the algorithm were calculated based on 2 by 2 tables, using the manual review of the EMR as reference the test. Each result of the detection algorithm was classified as true positive, false positive, true negative or false negative, both for CaVD and CeVD pathology separately, as well as a combined outcome. Analyses were carried out using SAS University Edition (SAS Institute Inc, USA).

### Qualitative analysis of errors

We qualitatively analyzed false positives and false negatives. The reviewers described the reasons that led them to classify each case as an error. These errors were then grouped and quantified in different subcategories.

## Results

### Quantitative analysis

**Fig. 1:**
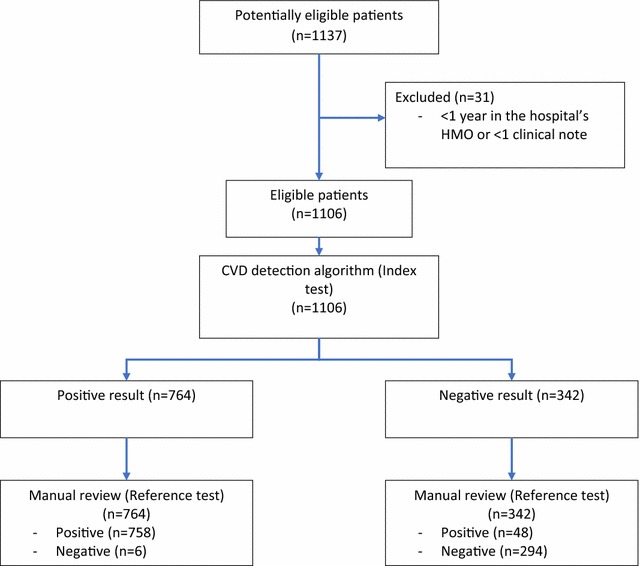
Flowchart

The sample was composed of 1137 patients. 31 did not fulfill the inclusion criteria. 1106 were included in the final analysis. See Fig. 1.

Table 1 shows the results of comparing the classification by the algorithm to the manual review. We can see that the kappa statistic, sensitivity and specificity show more than acceptable values (Table 2).

**Table 1:** Performance of the algorithm compared to manual review

Estimate (95% CI)	CaVD	CeVD	CaVD or CeVD
Sensitivity	96% (93.42–97.35)	97% (95.6–98.68)	99% (98.3–99.71)
Specificity	93% (90.91–94.98)	97% (94.79–97.81)	86% (81.83–89.47)
κ coefficient	0.88 (0.85–0.91)	0.94 (0.92–0.96)	0.88 (0.85–0.91)

*CaVD* cardiovascular disease, *CeVD* cerebrovascular disease

**Table 2:** Stratified sample of 1106 patients as classified by the algorithm

Presence of disease		n (%)
CaVD	CeVD	
Yes	No	324 (29.29)
No	Yes	318 (28.75)
Both present		164 (14.83)
Both absent		300 (27.12)

*CaVD* cardiovascular disease, *CeVD* cerebrovascular disease

### Qualitative analysis of errors

Erroneous results of the detection algorithm were classified into false positive (FP) and false negative (FN) results. They are described in Table 3. Table 4 describes the distribution of the erroneous results classified by type of clinical problem.

**Table 3:** Distribution of erroneous outcomes of the detection algorithm of cardiovascular disease or stroke through the records of an electronic medical record

False negative results (FN) n (%)	Proportion			
	Total errors (FP + FN) (n = 98)	Total FN (n = 32)	CaVD FN (n = 21)	CeVD FN (n = 11)
Codes not included in the algorithm	20 (20.41)	20 (62.5)	15 (71.43)	5 (45.45)
Event included in the free-text clinical notes, but not coded	9 (9.18)	9 (28.13)	5 (23.81)	4 (36.36)
Coded terms not detected	2 (2.04)	2 (6.25)	1 (4.76)	1 (9.09)
Erroneous date	1 (1.02)	1 (3.13)	0 (0)	1 (9.09)

False positive results (FP) n (%)	Proportion			
	Total errors (FP + FN) (n = 98)	Total FP (n = 66)	CaVD FP (n = 39)	CeVD FP (n = 27)
Dismissed diagnoses^a^	43 (43.88)	43 (65.15)	33 (84.61)	10 (37.04)
Incidental finding without clinical relevance	13 (13.27)	13 (19.7)	0 (0)	13 (48.15)
Coded terms without correlation in the clinical notes	8 (8.16)	8 (12.12)	4 (10.26)	4 (14.81)
Incorrect abbreviations	2 (2.04)	2 (3.03)	2 (5.13)	0 (0)

*FP* false positive result, *FN* false negative result, *CaVD* cardiovascular events, *CeVD* cerebrovascular events

^a^For example, patients in whom an acute myocardial infarction was suspected but later on the diagnosis was rejected due to new information

**Table 4:** Distribution of erroneous outcomes, classified by type of clinical problem

Results wrong depending on clinical problem n (%)	Proportion				
	Of the total number of errors (n = 98)	The fn (n = 32)	The FP (n = 66)	Errors	
				CaV (n = 62)	CeV (n = 36)
Peripheral vascular disease	18 (18.37)	10 (31.25)	8 (12.12)	18 (30)	NA
Heart failure	11 (11.22)	4 (12.5)	7 (10.61)	11 (18.33)	
Acute myocardial infarction	11 (11.22)	5 (15.63)	6 (9.09)	11 (18.33)	
Cardiovascular symptoms^a^	22 (22.45)	4 (12.5)	18 (27.27)	22 (36.67)	
Stroke/CeVD	29 (29.6)	2 (6.25)	27 (40.91)	NA	29 (81.58)
Transient ischemic attack	7 (7.14)	7 (21.87)	0 (0)		7 (18.42)

*NA* not applicable

^a^Chest pain, angina pectoris, chest oppression

## Discussion

This algorithm was developed in the context of constructing a retrospective cohort of patients without CeVD or CaVD, emulating the exclusion criteria of the cohorts used for the development of several cardiovascular risk calculators [51, 52]. For this purpose, the sensitivity and specificity achieved seem more than appropriate. However, it is important to note that when using the combined algorithm to exclude patients with history of CaVD and CeVD, its lower specificity might create a cohort that is slightly healthier than the average population of the HIBA.

The qualitative analysis of errors provides relevant information regarding the algorithm itself, the EMR and also about the physicians’ EMR usage habits. The high sensitivity of the algorithm is probably due to the relevance of the selected problems; we did not expect to find underreporting or “undercoding”^1^ of events such as MI, CHF or stroke that are both clinically relevant and usually easier to define than other subtler entities. Nevertheless, problems that tend to be harder to define like CaVD symptoms or PVD represented an important fraction of the total FPs.

The lower specificity found required an analysis of the FPs. The most common type of error was the dismissal of a diagnostic code. This was usually related to not deleting or registering the code as an error once further testing had dismissed the initial diagnosis. The EMR of the HIBA allows assigning each code, a status (active, passive or error). Active codes or problems are those that the treating physicians deems as a current confirmed problem that requires an intervention. Passive problems are those that although confirmed, do not require an intervention at the moment (i.e. stable, well controlled hypertension). The status ‘Error’ is assigned to those codes that prove to be false, usually after further testing. The results of our study seem to indicate that many diagnoses initially coded as active, once they are later on dismissed (mainly observed in cardiovascular and PVP symptoms), their status is not changed to error. This could be improved through interventions on how physicians interact with the EMR or, from the algorithm point of view, by implementing natural language processing (NLP) algorithms to obtain information from the free text. However, this would add a new level of complexity to the algorithm that might not be cost-effective given the costly process involved in training NLP tools.

The second most frequent cause of FP are codes that do not translate to clinically relevant events, for example the incidental finding of lacunar lesions in a brain CT scan that was requested not due to acute neurological symptoms, but rather due to head trauma or cognitive impairment. This could also be improved by implementing the strategies mentioned in the previous paragraph.

It is difficult to find an explanation regarding the coded terms for which no information was found in the free text. They could simply be recording errors. Nevertheless, given the fact that some of these were very relevant terms, like stroke or MI, we believe that there must be some alterative explanation that is eluding us.

One of our main goals was to develop a parsimonious algorithm that required the least amount of information to be able to correctly classify patients. In this sense, other authors have also used coded terms to develop algorithms for detection of cases with good results. Regarding CHF, Kottke et al. developed an algorithm with excellent sensitivity and specificity only based on ICD codes [43]. The results are consistent with the review carried out by Saczynski and cols [50]. In MI and stroke/TIA similar results were found [13, 17, 44]. More complex algorithms, which for example include laboratory results or imaging reports, did not perform superiorly than those only based on codes, at least for these pathologies [13, 44]. Nevertheless, as shown by Fan and cols [11], algorithms that include more complex and data-driven techniques, like NLP or regression techniques, seem to perform better. On the other hand, algorithms based only on data driven processes can be less generalizable over time even within a single institution, given the usual changes in language throughout time.

Detecting many pathologies using a single algorithm is useful for the design of studies requiring broad exclusion/inclusion criteria. In this respect, we found only a few previous attempts to do so in the literature. Tu et al. describe an algorithm to detect cases of stroke/TIA combined; Kottke et al. do the same for MI and CHF [13]. Our algorithm could contribute to fill this information gap.

One of the limitations of this study is that by not limiting the algorithm to standardized codes, it might be too specific for our hospital. However, this could also be seen as a strength: perhaps the algorithms that have better performance are precisely those that are developed locally by people who have a profound knowledge of the idiosyncrasies of the EMR users and thus incorporate nonstandard local terms.

On the other hand, it is important to emphasize that this algorithm sought to emulate the medical criteria for the detection of cases and not the detection of true cases, since no objective criteria were used in the algorithm or the manual review process. Therefore, if the physician made a mistake in the diagnosis process, this would be not detected by the algorithm.

Finally, these results may not be generalizable within our hospital given that we only evaluated charts until 2005 and not later.

## Conclusion

We developed a simple algorithm, using only standardized and non-standardized coded terms within an EMR that can properly detect clinically relevant cases of CaVD and CeVD with excellent sensitivity. If used to rule in cases, manual review of the detected cases might be granted because of the algorithm’s lower specificity. Nevertheless, we believe that the specificity level is difficult to improve without altering the excellent sensitivity attained, since much of the deficit of specificity is due how users interact with the EMR. We believe that this would hardly be rectified by the inclusion or exclusion of terms in the algorithm, but perhaps combining it with an analysis of the free text using an NLP approach would yield better results.

## Additional file

**Additional file 1: Appendix.**List of terms included in the algorithm

## Footnotes

1 Not coding a medical condition, diagnosis or procedure, but including it in the free text of the clinical note.

## References

[1] Kukafka R (2007). Redesigning electronic health record systems to support public health. J Biomed Inform.

[2] Chaudhry B (2006). Systematic review: impact of health information technology on quality, efficiency, and costs of medical care. Ann Intern Med.

[3] Afzal Z (2013). Automatic generation of case-detection algorithms to identify children with asthma from large electronic health record databases. Pharmacoepidemiol Drug Saf.

[4] Schuemie MJ (2012). Automating classification of free-text electronic health records for epidemiological studies. Pharmacoepidemiol Drug Saf.

[5] Valkhoff VE (2014). Validation study in four health-care databases: upper gastrointestinal bleeding misclassification affects precision but not magnitude of drug-related upper gastrointestinal bleeding risk. J Clin Epidemiol.

[6] Jensen PB, Jensen LJ, Brunak S (2012). Mining electronic health records: towards better research applications and clinical care. Nat Rev Genet.

[7] Ho ML (2012). The accuracy of using integrated electronic health care data to identify patients with undiagnosed diabetes mellitus. J Eval Clin Pract.

[8] Klompas M (2013). Automated detection and classification of type 1 versus type 2 diabetes using electronic health record data. Diabetes Care.

[9] Kudyakov R (2012). Electronic health record use to classify patients with newly diagnosed versus preexisting type 2 diabetes: infrastructure for comparative effectiveness research and population health management. Popul Health Manag.

[10] Lawrence JM (2014). Validation of pediatric diabetes case identification approaches for diagnosed cases by using information in the electronic health records of a large integrated managed health care organization. Am J Epidemiol.

[11] Fan J (2013). Billing code algorithms to identify cases of peripheral artery disease from administrative data. J Am Med Inform Assoc.

[12] Hammad TA (2008). Determining the predictive value of Read/OXMIS codes to identify incident acute myocardial infarction in the General Practice Research Database. Pharmacoepidemiol Drug Saf.

[13] Kottke TE, Baechler CJ. An algorithm that identifies coronary and heart failure events in the electronic health record. Prev Chronic Dis. 2013;10:E29. doi:10.5888/pcd10.120097. 10.5888/pcd10.120097 PMC3592787 23449283

[14] Murff HJ (2011). Automated identification of postoperative complications within an electronic medical record using natural language processing. JAMA.

[15] Fleet JL (2013). Detecting chronic kidney disease in population-based administrative databases using an algorithm of hospital encounter and physician claim codes. BMC Nephrol.

[16] Dregan A (2011). Utility of electronic patient records in primary care for stroke secondary prevention trials. BMC Public Health.

[17] Tu K (2013). Validity of administrative data for identifying patients who have had a stroke or transient ischemic attack using EMRALD as a reference standard. Can J Cardiol.

[18] Churpek MM (2014). Using electronic health record data to develop and validate a prediction model for adverse outcomes in the wards*. Crit Care Med.

[19] Fan J (2013). Billing code algorithms to identify cases of peripheral artery disease from administrative data. J Am Med Inform Assoc.

[20] Jensen PN (2012). A systematic review of validated methods for identifying atrial fibrillation using administrative data. Pharmacoepidemiol Drug Saf.

[21] Kadhim-Saleh A (2013). Validation of the diagnostic algorithms for 5 chronic conditions in the Canadian Primary Care Sentinel Surveillance Network (CPCSSN): a Kingston Practice-based Research Network (PBRN) report. J Am Board Fam Med.

[22] Vijayakrishnan R (2014). Prevalence of heart failure signs and symptoms in a large primary care population identified through the use of text and data mining of the electronic health record. J Card Fail.

[23] Savova GK (2008). Mayo clinic NLP system for patient smoking status identification. J Am Med Inform Assoc.

[24] Sohn S, Savova GK (2009). Mayo clinic smoking status classification system: extensions and improvements. AMIA Annu Symp Proc.

[25] Uzuner O (2008). Identifying patient smoking status from medical discharge records. J Am Med Inform Assoc.

[26] Wu CY (2013). Evaluation of smoking status identification using electronic health records and open-text information in a large mental health case register. PLoS ONE.

[27] Hivert MF (2009). Identifying primary care patients at risk for future diabetes and cardiovascular disease using electronic health records. BMC Health Serv Res.

[28] Alsara A (2011). Derivation and validation of automated electronic search strategies to identify pertinent risk factors for postoperative acute lung injury. Mayo Clin Proc.

[29] Green BB (2012). Using body mass index data in the electronic health record to calculate cardiovascular risk. Am J Prev Med.

[30] Persell SD (2009). Electronic health record-based cardiac risk assessment and identification of unmet preventive needs. Med Care.

[31] Richards A, Cheng EM (2013). Stroke risk calculators in the era of electronic health records linked to administrative databases. Stroke.

[32] Deleger L, Grouin C, Zweigenbaum P (2010). Extracting medical information from narrative patient records: the case of medication-related information. J Am Med Inform Assoc.

[33] Fung KW, Jao CS, Demner-Fushman D (2013). Extracting drug indication information from structured product labels using natural language processing. J Am Med Inform Assoc.

[34] Levin MA (2007). Extraction and mapping of drug names from free text to a standardized nomenclature. AMIA Annu Symp Proc.

[35] Xu H (2010). MedEx: a medication information extraction system for clinical narratives. J Am Med Inform Assoc.

[36] Sai K (2013). Development of a detection algorithm for statin-induced myopathy using electronic medical records. J Clin Pharm Ther.

[37] Skentzos S (2011). Structured vs. unstructured: factors affecting adverse drug reaction documentation in an EMR repository. AMIA Annu Symp Proc.

[38] Suissa S, Garbe E (2007). Primer: administrative health databases in observational studies of drug effects–advantages and disadvantages. Nat Clin Pract Rheumatol.

[39] Coloma PM (2011). Combining electronic healthcare databases in Europe to allow for large-scale drug safety monitoring: the EU-ADR Project. Pharmacoepidemiol Drug Saf.

[40] Coloma PM (2013). Drug-induced acute myocardial infarction: identifying ‘prime suspects’ from electronic healthcare records-based surveillance system. PLoS ONE.

[41] Assaf AR (2000). Coronary heart disease surveillance: field application of an epidemiologic algorithm. J Clin Epidemiol.

[42] Cutrona SL (2013). Validation of acute myocardial infarction in the Food and Drug Administration’s Mini-Sentinel program. Pharmacoepidemiol Drug Saf.

[43] Kottke TE, Baechler CJ, Parker ED (2012). Accuracy of heart disease prevalence estimated from claims data compared with an electronic health record. Prev Chronic Dis.

[44] Tu K (2010). Validation of physician billing and hospitalization data to identify patients with ischemic heart disease using data from the Electronic Medical Record Administrative data Linked Database (EMRALD). Can J Cardiol.

[45] Gulliford MC (2009). Selection of medical diagnostic codes for analysis of electronic patient records. Application to stroke in a primary care database. PLoS ONE.

[46] Allen LA (2014). Performance of claims-based algorithms for identifying heart failure and cardiomyopathy among patients diagnosed with breast cancer. Med Care.

[47] Loehr LR (2013). Classification of acute decompensated heart failure: an automated algorithm compared with a physician reviewer panel: the Atherosclerosis Risk in Communities study. Circ Heart Fail.

[48] Lee DS (2005). Comparison of coding of heart failure and comorbidities in administrative and clinical data for use in outcomes research. Med Care.

[49] Rosenman M (2014). Database queries for hospitalizations for acute congestive heart failure: flexible methods and validation based on set theory. J Am Med Inform Assoc.

[50] Saczynski JS (2012). A systematic review of validated methods for identifying heart failure using administrative data. Pharmacoepidemiol Drug Saf.

[51] Karmali KN (2014). A systematic examination of the 2013 ACC/AHA pooled cohort risk assessment tool for atherosclerotic cardiovascular disease. J Am Coll Cardiol.

[52] D’Agostino RB (2001). Validation of the Framingham coronary heart disease prediction scores: results of a multiple ethnic groups investigation. JAMA.

