# Supplementary material for: A rule-based electronic phenotyping algorithm for detecting clinically relevant cardiovascular disease cases
Source: BMC Res Notes. 2017 Jul 14;10:281. doi: 10.1186/s13104-017-2600-2 (PMC5513369; doi:10.1186/s13104-017-2600-2)
Supplement: Supplementary file 1 — Additional file 1: Appendix.List of terms included in the algorithm [file 13104_2017_2600_MOESM1_ESM.docx]

1. **Appendix**
   1. **SNOMED-CT codes included in the final algorithm**

| SNOMED Reference ID | SNOMED CT Name |
| --- | --- |
| 57054005 | Acute myocardial infarction (disorder) |
| 233841005 | Old lateral myocardial infarction (disorder) |
| 233839009 | Old anterior myocardial infarction (disorder) |
| 1755008 | Old myocardial infarction (disorder) |
| 233840006 | Old inferior myocardial infarction (disorder) |
| 233829003 | Acute Q wave infarction - inferior (disorder) |
| 73795002 | Acute myocardial infarction of inferior wall (disorder) |
| 307140009 | Acute non-Q wave infarction (disorder) |
| 54329005 | Acute myocardial infarction of anterior wall (disorder) |
| 89138009 | Cardiogenic shock (disorder) |
| 58612006 | Acute myocardial infarction of lateral wall (disorder) |
| 401303003 | Acute ST segment elevation myocardial infarction (disorder) |
| 22298006 | Myocardial infarction (disorder) |
| 398274000 | Coronary artery thrombosis (disorder) |
| 76388001 | ST segment elevation (finding) |
| 394659003 | Acute coronary syndrome (disorder) |
| 76593002 | Acute myocardial infarction of inferoposterior wall (disorder) |
| 233843008 | Silent myocardial infarction (disorder) |
| 304914007 | Acute Q wave myocardial infarction (disorder) |
| 52035003 | Acute anteroapical myocardial infarction (disorder) |
| 70211005 | Acute myocardial infarction of anterolateral wall (disorder) |
| 233828006 | Acute non-Q wave infarction - anterolateral (disorder) |
| 233827001 | Acute Q wave infarction - anterolateral (disorder) |
| 62695002 | Acute anteroseptal myocardial infarction (disorder) |
| 90539001 | Ventricular aneurysm (disorder) |
| 84114007 | Heart failure (disorder) |
| 42343007 | Congestive heart failure (disorder) |
| 65547006 | Acute myocardial infarction of inferolateral wall (disorder) |
| 233838001 | Acute posterior myocardial infarction (disorder) |
| 79009004 | Acute myocardial infarction of septum (disorder) |
| 59063002 | Acute myocardial infarction of apical-lateral wall (disorder) |
| 314116003 | Post infarct angina (disorder) |
| 233834004 | Acute non-Q wave infarction - lateral (disorder) |
| 15990001 | Acute myocardial infarction of posterolateral wall (disorder) |
| 233830008 | Acute non-Q wave infarction - inferior (disorder) |
| 59931005 | Inverted T wave (finding) |
| 70422006 | Acute subendocardial infarction (disorder) |
| 25106000 | Impending infarction (disorder) |
| 20529002 | Secondary dilated cardiomyopathy (disorder) |
| 194866002 | Rupture of chordae tendinae as current complication following acute myocardial infarction (disorder) |
| 233847009 | Cardiac rupture after acute myocardial infarction (disorder) |
| 253541009 | Left ventricular dilatation (disorder) |
| 194856005 | Subsequent myocardial infarction (disorder) |
| 161640005 | High risk of heart disease (finding) |
| 4557003 | Preinfarction syndrome (disorder) |
| 401314000 | Acute non-ST segment elevation myocardial infarction (disorder) |
| 71884009 | Precordial pain (finding) |
| 314207007 | Non-Q wave myocardial infarction (disorder) |
| 233832000 | Acute non-Q wave infarction - inferolateral (disorder) |
| 414545008 | Ischemic heart disease (disorder) |
| 53741008 | Coronary arteriosclerosis (disorder) |
| 27550009 | Disorder of blood vessel (disorder) |
| 371803003 | Multi vessel coronary artery disease (disorder) |
| 399211009 | History of myocardial infarction (situation) |
| 233819005 | Stable angina (disorder) |
| 91335003 | Mural thrombus of heart (disorder) |
| 40541001 | Acute pulmonary edema (disorder) |
| 66189004 | Postmyocardial infarction syndrome (disorder) |
| 26141007 | ST segment depression (finding) |
| 44103008 | Ventricular arrhythmia (disorder) |
| 195239002 | Late effects of cerebrovascular disease (disorder) |
| 415992005 | Disorder of right cardiac ventricle (disorder) |
| 414024009 | Disorder of coronary artery (disorder) |
| 282006 | Acute myocardial infarction of basal-lateral wall (disorder) |
| 70998009 | Acute myocardial infarction of posterobasal wall (disorder) |
| 64627002 | Acute myocardial infarction of high lateral wall (disorder) |
| 3098007 | Rupture of interventricular septum (disorder) |
| 230690007 | Cerebrovascular accident (disorder) |
| 230715005 | Posterior circulation stroke of uncertain pathology (disorder) |
| 413758000 | Cardioembolic stroke (disorder) |
| 195217004 | Right sided cerebral hemisphere cerebrovascular accident (disorder) |
| 195216008 | Left sided cerebral hemisphere cerebrovascular accident (disorder) |
| 422504002 | Ischemic stroke (disorder) |
| 111297002 | Nonparalytic stroke (disorder) |
| 373606000 | Occlusive stroke (disorder) |
| 116288000 | Paralytic stroke (disorder) |
| 371041009 | Embolic stroke (disorder) |
| 371040005 | Thrombotic stroke (disorder) |
| 87486003 | Aphasia (finding) |
| 229665008 | Expressive dysphasia (finding) |
| 128609009 | Intracranial aneurysm (disorder) |
| 230801000 | Post-ictal coma (disorder) |
| 264552009 | Neurological deficit (finding) |
| 429998004 | Vascular dementia (disorder) |
| 8011004 | Dysarthria (finding) |
| 62914000 | Cerebrovascular disease (disorder) |
| 288723005 | Acute ill-defined cerebrovascular disease (disorder) |
| 301764006 | Hematoma of brain (disorder) |
| 77674003 | Hemianopia (finding) |
| 278286009 | Right hemiparesis (disorder) |
| 278287000 | Left hemiparesis (disorder) |
| 50582007 | Hemiplegia (disorder) |
| 278284007 | Right hemiplegia (disorder) |
| 278285008 | Left hemiplegia (disorder) |
| 75038005 | Cerebellar hemorrhage (disorder) |
| 274100004 | Cerebral hemorrhage (disorder) |
| 195167002 | External capsule hemorrhage (disorder) |
| 73020009 | Cerebral hemisphere hemorrhage (disorder) |
| 195165005 | Basal ganglia hemorrhage (disorder) |
| 237702003 | Pituitary hemorrhage (disorder) |
| 276722003 | Intracerebellar and posterior fossa hemorrhage (disorder) |
| 1386000 | Intracranial hemorrhage (disorder) |
| 23276006 | Ventricular hemorrhage (disorder) |
| 428241007 | Ventricular hemorrhage of fetus (disorder) |
| 230712008 | Lacunar hemorrhage (disorder) |
| 7713009 | Intrapontine hemorrhage (disorder) |
| 194179009 | Retrobulbar hemorrhage (disorder) |
| 21454007 | Subarachnoid intracranial hemorrhage (disorder) |
| 230719004 | Intracranial subarachnoid hemorrhage due to ruptured aneurysm (disorder) |
| 276278003 | Subarachnoid hemorrhage from anterior cerebral artery aneurysm (disorder) |
| 276284000 | Subarachnoid hemorrhage from basilar artery aneurysm (disorder) |
| 276280009 | Subarachnoid hemorrhage from middle cerebral artery aneurysm (disorder) |
| 276281008 | Subarachnoid hemorrhage from posterior cerebral artery aneurysm (disorder) |
| 276282001 | Subarachnoid hemorrhage from anterior communicating artery aneurysm (disorder) |
| 276283006 | Subarachnoid hemorrhage from posterior communicating artery aneurysm (disorder) |
| 230711001 | Thalamic hemorrhage (disorder) |
| 49453006 | Cerebral herniation (disorder) |
| 38341003 | Hypertensive disorder, systemic arterial (disorder) |
| 95460007 | Cerebellar infarction (disorder) |
| 432504007 | Cerebral infarction (disorder) |
| 230706003 | Hemorrhagic cerebral infarction (disorder) |
| 276219001 | Occipital cerebral infarction (disorder) |
| 195190007 | Cerebral infarction due to embolism of cerebral arteries (disorder) |
| 195186005 | Cerebral infarction due to embolism of precerebral arteries (disorder) |
| 195189003 | Cerebral infarction due to thrombosis of cerebral arteries (disorder) |
| 195185009 | Cerebral infarct due to thrombosis of precerebral arteries (disorder) |
| 195230003 | Cerebral infarction due to cerebral venous thrombosis, non-pyogenic (disorder) |
| 95457000 | Brain stem infarction (disorder) |
| 230707007 | Anterior cerebral circulation hemorrhagic infarction (disorder) |
| 230708002 | Posterior cerebral circulation hemorrhagic infarction (disorder) |
| 230698000 | Lacunar infarction (disorder) |
| 230699008 | Pure motor lacunar infarction (disorder) |
| 230694003 | Total anterior cerebral circulation infarction (disorder) |
| 307363008 | Multiple lacunar infarcts (disorder) |
| 233957006 | Arterial ischemia (disorder) |
| 72986009 | Acute hemorrhagic leukoencephalitis (disorder) |
| 253193002 | Vascular malformation of the nervous system (disorder) |
| 77291009 | Complete unilateral paresis (situation) |
| 102568007 | Paresis of lower extremity (finding) |
| 293331003 | Anticoagulant adverse reaction (disorder) |
| 233983001 | Ruptured cerebral aneurysm (disorder) |
| 277316004 | Ruptured aneurysm of middle cerebral artery (disorder) |
| 277299009 | Ruptured cerebral arteriovenous malformation (disorder) |
| 195213000 | Cerebellar stroke syndrome (disorder) |
| 195212005 | Brainstem stroke syndrome (disorder) |
| 425882004 | Paralytic syndrome as late effect of stroke (disorder) |
